# Vitamin D in Cutaneous T-Cell Lymphoma

**DOI:** 10.3390/cells13060503

**Published:** 2024-03-13

**Authors:** August-Witte Feentved Ødum, Carsten Geisler

**Affiliations:** The LEO Foundation Skin Immunology Research Center, Department of Immunology and Microbiology, Faculty of Health and Medical Sciences, University of Copenhagen, DK-2200 Copenhagen, Denmark

**Keywords:** CTCL, vitamin D, pathogenesis, treatment

## Abstract

Cutaneous T-cell lymphoma (CTCL) is characterized by the proliferation of malignant T cells in inflamed skin lesions. Mycosis fungoides (MF)—the most common variant of CTCL—often presents with skin lesions around the abdomen and buttocks (“bathing suit” distribution), i.e., in skin areas devoid of sun-induced vitamin D. For decades, sunlight and vitamin D have been connected to CTCL. Thus, vitamin D induces apoptosis and inhibits the expression of cytokines in malignant T cells. Furthermore, CTCL patients often display vitamin D deficiency, whereas phototherapy induces vitamin D and has beneficial effects in CTCL, suggesting that light and vitamin D have beneficial/protective effects in CTCL. Inversely, vitamin D promotes T helper 2 (Th2) cell specific cytokine production, regulatory T cells, tolerogenic dendritic cells, as well as the expression of immune checkpoint molecules, all of which may have disease-promoting effects by stimulating malignant T-cell proliferation and inhibiting anticancer immunity. Studies on vitamin D treatment in CTCL patients showed conflicting results. Some studies found positive effects, others negative effects, while the largest study showed no apparent clinical effect. Taken together, vitamin D may have both pro- and anticancer effects in CTCL. The balance between the opposing effects of vitamin D in CTCL is likely influenced by treatment and may change during the disease course. Therefore, it remains to be discovered whether and how the effect of vitamin D can be tilted toward an anticancer response in CTCL.

## 1. Sunlight, Vitamin D, and Cutaneous T-Cell Lymphoma

Vitamin D is a fat-soluble secosteroid that comes in two forms, namely vitamin D2 (ergocalciferol) and vitamin D3 (cholecalciferol). These forms differ only in the side chain, and the biological activities are comparable [1]. Vitamin D3 is produced in the skin from 7-dehydrocholesterol [2], whereas vitamin D2 is produced in plants and fungi from ergosterol [3]. For decades, it has been known that sunlight elicits significant production of vitamin D3 in the skin. Solar ultraviolet B (UVB) radiation (280–315 nm) is absorbed by 7-dehydrocholesterol in the plasma membrane of keratinocytes, leading to its transformation to pre-vitamin D3, which is rapidly converted to vitamin D3 [4,5,6,7]. Vitamin D3 is ejected from the plasma membrane into the extracellular space and then drawn into the circulation by the vitamin D binding protein (DBP), which has high affinity for vitamin D3 [6,8]. Vitamin D3 is transported by DBP to the liver, where it is metabolized to 25(OH)D3 (calcifediol) by vitamin D 25-hydroxylases CYP2R1 and CYP27A1 [7]. 25(OH)D3 re-enters the circulation, where it is bound to DBP. In the kidney, 25(OH)D3 is converted to the active form of vitamin D, 1,25(OH)_2_D3 (calcitriol), by the 25(OH)D 1-α hydroxylase CYP27B1 [9,10,11].

Humans have a combination of vitamins D2 and D3 available to them derived from UV-exposed skin (vitamin D3), dietary intakes of vitamin D3-rich foods such as egg yolks and oily fish, fortified foods that generally have vitamin D2 fortification, and vitamin supplements that are available both as vitamins D2 and D3 [12]. The concentration of 25(OH)D (25(OH)D2 plus 25(OH)D3 (from here on D is D2 plus D3) in serum is considered the best parameter for the evaluation of the vitamin D status of a subject. Although the reliance on a single cutoff value to define vitamin D deficiency or insufficiency is problematic because of the wide individual variability of the functional effects of vitamin D [13,14], the Endocrine Society defines vitamin D deficiency as a 25(OH)D concentration below 50 nM (20 ng/mL) and vitamin D insufficiency as a 25(OH)D concentration of 51–72 nM (21–29 ng/mL) [12]. There is consensus that severe vitamin D deficiency (serum 25(OH)D concentration < 30 nM) should be corrected, whereas most guidelines recommend serum 25(OH)D concentrations of >50 nM for optimal bone health in older adults [15]. However, the causal link between vitamin D and many extra-skeletal outcomes remains unclear. The serum concentration of the active 1,25(OH)_2_D is approximately 1000-fold lower (60–110 pM, 25–46 pg/mL) than the 25(OH)D concentration, far below the effective concentration of 1,25(OH)_2_D applied in the majority of in vitro studies on the effects of vitamin D on immune cells. Thus, more than a 100-fold higher concentration of 1,25(OH)_2_D than the amount found in serum is often required to obtain an effect in most in vitro studies [16,17,18,19,20,21,22]. It has therefore been suggested that the level of circulating 1,25(OH)_2_D is too low to affect immune responses in vivo, and that sufficient levels are obtained by the local conversion of 25(OH)D to 1,25(OH)_2_D [23,24,25]. Accordingly, a number of other tissues than the kidney also express CYP27B1 and can produce 1,25(OH)_2_D [7,26]. 

In the skin, keratinocytes, dendritic cells (DCs), and macrophages express CYP27B1 and have the capacity to produce 1,25(OH)_2_D [11,25,26,27]. The production of 1,25(OH)_2_D in the skin could serve in regulating skin resident cells, including keratinocytes, immune cells, and, in special cases, malignant cells. Cutaneous T-cell lymphoma (CTCL) is characterized by the proliferation of malignant T cells in inflamed skin lesions that potentially could be affected by vitamin D. The most common variant of CTCL, mycosis fungoides (MF), often presents with lesions in skin areas devoid of sun-induced vitamin D around the abdomen and buttocks, the so-called “bathing suit” distribution. The characteristic distribution of MF has contributed to the idea that sunlight and vitamin D might have a preventive effect against CTCL [28,29,30]. The notion was further accentuated by the recent discovery of an association between vitamin D deficiency and the risk of disease progression in other types of lymphoma [31,32,33] and a recent case study on juvenile CTCL and hypovitaminosis D [34].

## 2. The Vitamin D Receptor

**Figure 1:**
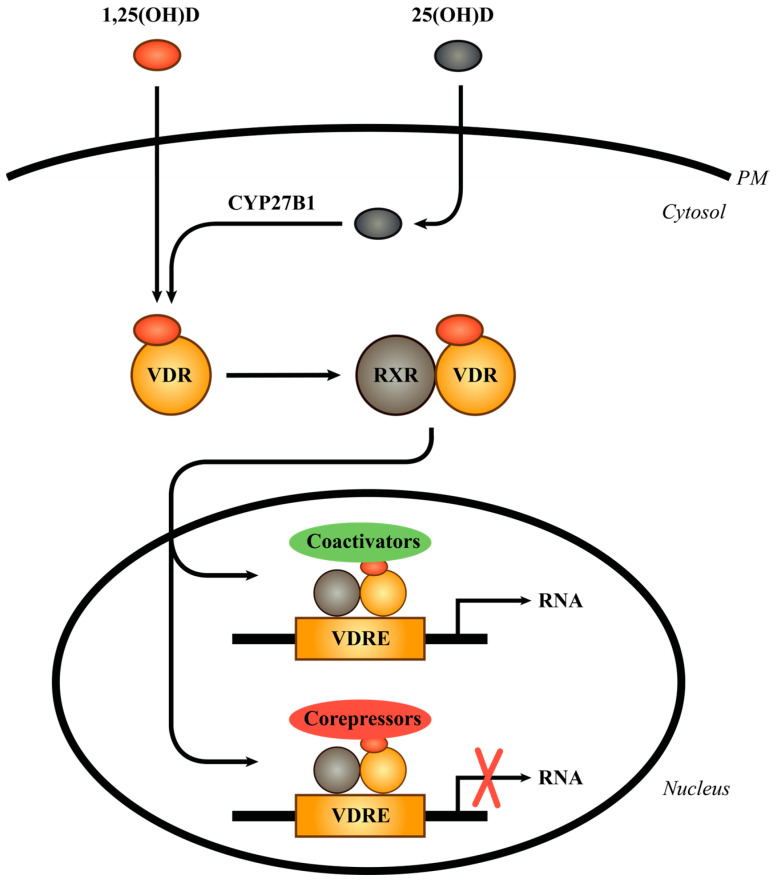
Simplified overview of the vitamin D/VDR signaling pathway. PM, plasma membrane; VDR, vitamin D receptor; RXR, retinoid X receptor; VDRE, vitamin D response element.

The cellular actions of 1,25(OH)_2_D are mediated by the vitamin D receptor (VDR), a ligand-dependent transcription factor belonging to the superfamily of nuclear receptors [35,36]. Following interaction with 1,25(OH)_2_D, the VDR dimerizes with the retinoid X receptor (RXR) and translocates to the nucleus, where it binds to vitamin D response elements (VDREs) in vitamin D responsive genes. Depending on the target gene, either coactivators or corepressors are attracted to the 1,25(OH)_2_D/VDR/RXR complexes to induce or repress gene transcription. Figure 1 gives a simplified overview of the vitamin D/VDR signaling pathway. For a more detailed description of vitamin D/VDR-mediated gene regulation, please see [10,37,38,39,40,41,42]. The highest concentration of VDR is found in the intestine, kidney, and bone involved in the maintenance of calcium homeostasis. However, the VDR has been found in many other tissues, including the skin, not involved in calcium homeostasis, suggesting that vitamin D affects many cellular processes beyond the regulation of calcium homeostasis [9,11,43].

## 3. Effect of 1,25(OH)_2_D on Cells in the Skin

Many skin cells, including keratinocytes and immune cells, express the VDR [9,11,44]. Acting through the VDR, 1,25(OH)_2_D regulates the proliferation of the basal layer of the epidermis and the sequential differentiation of keratinocytes [2,11,44,45,46]. The loss of VDR disrupts keratinocyte differentiation and epithelial barrier formation, impairs wound healing, and predisposes to cancer [47,48,49,50,51].

It has long been recognized that 1,25(OH)_2_D plays an important role in the regulation and function of the immune system [52,53,54,55]. Accordingly, various immune cells express the VDR [52,55,56]. 1,25(OH)_2_D affects the activation and differentiation of several types of immune cells via the VDR [57]. Among other effects, it promotes the differentiation of monocytes into macrophages and the expression of the antimicrobial peptides cathelicidin and beta-defensin [40,53,58]. In this way, 1,25(OH)_2_D plays an important role in the immune system’s defense against bacteria and in particular mycobacteria [40,53,58]. In support, patients with genetic defects in the VDR have a compromised cathelicidin response and increased susceptibility to infection with *Mycobacterium tuberculosis* and other mycobacteria [53,59]. 1,25(OH)_2_D has recently been ascribed a role in COVID-19 infections, but the clinical importance remains controversial [60]. In addition to its role in the antimicrobial defense, 1,25(OH)_2_D has also anti-inflammatory effects such as increasing the tolerogenic effect of macrophages and other antigen-presenting cells. This is achieved partly through the inhibition of tumor necrosis factor α (TNF-α) and interleukin (IL)-6 secretion, as well as a decreased expression of Toll-like receptor (TLR) molecules and the class II major histocompatibility complex (MHC II). Furthermore, 1,25(OH)_2_D can also promote a change toward a tolerogenic phenotype in DC by increasing the expression of inhibitory molecules such as programmed death ligand-1 (PD-L1) [61]. In this way, tolerogenic DC induces T-cell anergy and induction of tolerogenic cytokines (IL-10), i.e., a phenotype resembling that of DC in cutaneous T-cell lymphoma (CTCL) [62].

In the skin, T cells play important roles in protective immunity and the development of inflammatory and autoimmune diseases [63,64,65]. Several studies have examined the role of 1,25(OH)_2_D and the VDR in T-cell development, differentiation, and function. Studies of mice lacking the VDR and in humans with a defect VDR showed a normal number of CD4^+^ and CD8^+^ T cells including naturally occurring CD4^+^FoxP3^+^ regulatory T cells [66,67,68], suggesting that the 1,25(OH)_2_D and VDR are not required for the development of either of these T-cell types. However, other studies indicated that 1,25(OH)_2_D is required for the normal development and function of invariant natural killer T cells [69] and CD8αα^+^ intraepithelial lymphocytes [68]. Naïve T cells express no or very low levels of the VDR; however, T-cell activation leads to the rapid upregulation of the VDR [24,56,70,71]. In accordance, 1,25(OH)_2_D significantly affects T-cell function [53,72,73]. In CD4^+^ T cells, for instance, VDR ligation leads to the inhibition of interferon (IFN)γ, IL-17A, and IL-22 expression, while it results in an increased expression of IL-4, IL-5, and IL-13 characteristic of T helper cell type 2 (Th2) responses [24,25,52,53,74,75]. In parallel, 1,25(OH)_2_D favors the expansion of regulator T cells (Treg) and the expression of the regulatory cytokine IL-10, suggesting that 1,25(OH)_2_D has an additional role in protecting against overt immune reactions [16,52,53,55,76].

Strikingly, solar UV radiation was shown to induce immunosuppression, inhibiting sensibilization in a model of contact hypersensitivity induced by an epicutaneous application of contact allergens [77,78,79]. Soon, it became clear that UV-induced immunosuppression was antigen-specific and mediated by Treg cells, tolerogenic antigen-presenting cells, and IL-10 [80,81,82], and later, it turned out that a multitude of cell types and cytokines—both in the innate and adaptive immune systems as well as in skin stroma cells—were affected by sunlight and involved in the complex immunological events following exposure to UV radiation [81,83]. Keratinocytes—the most abundant cell type in the epidermis—respond to UV radiation in a multitude of ways that directly and indirectly stimulate inflammation, immunosuppression, and DNA damage repair [84]. In addition to vitamin D, keratinocytes synthesize and release other effector molecules, including cytokines and enzymes, following exposure to UVB radiation [84,85].

## 4. Overview of Cutaneous T-Cell Lymphoma

CTCL belongs to a type of lymphoma collectively called non-Hodgkin’s lymphoma, which is often subdivided into four subgroups: Sezary syndrome (SS), a severe leukemic variant; CD30 positive lymphoproliferative diseases; mycosis fungoides (MF); and finally non-MF variants [86]. A characteristic feature of CTCL is epidermotropism—i.e., the accumulation of malignant T cells to the outermost layer of the skin that is potentially exposed to UV light [87]. CTCL often remains limited to the skin over long periods of disease evolution, and progression with extra-cutaneous spread usually occurs only in advanced late stages.

The most common variant of CTCL, MF, displays a characteristic disease evolution initially with erythematous patches (the patch stage), which may evolve into more infiltrated plaques (the plaque stage) [86]. The patches and plaques usually persist over a long time, but in a subset of patients, the disease develops into large and often ulcerated tumors (the tumor stage) or generalized erythroderma. Skin lesions may be associated with severe pruritus. The prognosis in the patch and limited plaque stage is favorable, whereas the tumor stage and generalized erythroderma MF are aggressive and associated with a risk of spreading outside the skin (extra-cutaneous MF). The type and extent of skin involvement as well as the presence of extra-cutaneous disease are significant prognostic factors [86] guiding the selection and type of therapeutic approaches and the acceptable risk of side effects [88].

Except for allogeneic stem cell transplantation and localized radiotherapy in unilesional MF, there are no curative therapies [89,90]. The treatment of MF initially involves skin-directed therapies (phototherapy/photochemotherapy, topical corticosteroids, and nitrogen mustard), which are the main strategies for early-stage MF. In subjects with later-stage disease, both approved and unapproved agents, including immune modulators, single agent, or combination chemotherapy (methotrexate, retinoids, pegylated interferon, electrophoresis, targeted therapies, and whole-body electron irradiation) are used. Potentially curative treatments are usually only relevant in subpopulations of patients. For example, allogeneic stem cell transplantation is usually only suitable for younger and fit patients with severe disease [89,90,91], whereas most treatment regimens are stage-directed and mainly directed at relieving symptoms and comorbidity. Accordingly, there is an unmet medical need for novel adjuvant therapies that can be combined with and amplify the effect of current forms of treatment.

The etiology of CTCL is largely unknown, and the pathogenesis is far from understood, but both genetic, epigenetic, and environmental factors are believed to be involved [91,92,93,94,95,96]. Disease heterogeneity is a characteristic feature of CTCL [93,97,98,99,100,101] even at the single-cell level [100,102,103,104,105], which has hampered the unraveling of the etiology and pathogenesis and has been a complicating factor in the development of novel therapies. Although high sun exposure is associated with a lower risk of developing MF in Caucasians [106], and heliotherapy (the use of natural sunlight for the treatment—also named climate therapy) [107,108] and phototherapy/photochemotherapy have beneficial effects on CTCL skin lesions [84,109], other studies indicate that UV light is also a driver of mutations in CTCL [110,111,112,113]. Thus, a large part of the mutational burden in MF and SS was linked to a UV signature, which was also observed in CD4^+^ T cells isolated from the blood of SS patients [111,112,113]. In support, some data indicated an association between occupational sun exposure and MF [110]. Interestingly, abnormally strong skin reactions to UVA and UVB were observed in a third of MF patients, suggesting that an increased sensitivity to UV light could play a pathogenic role in MF [114]. Taken together, these findings suggest that sunlight and UV radiation play complex—even opposing—roles in the initiation/aggravation versus protection/inhibition of the disease. Given the great heterogeneity among CTCL patients, it is also possible that synthesis, turnover, and responses to vitamin D—and its role in pathogenesis—are influenced by disease heterogeneity.

Numerous studies have consistently shown that infections are prevalent and significantly contribute to the morbidity and mortality of CTCL, especially among patients in advanced stages [90,115,116,117,118,119,120,121,122]. In a Danish twin study, susceptibility to severe bacterial infections in CTCL patients was not genetically determined but rather a vulnerability, which developed gradually after the diagnosis had been established [123].

Recent research supports the idea that malignant T cells orchestrate changes in the tumor microenvironment (TME), resulting in the suppression of skin barrier proteins like filaggrin, thereby compromising the skin barrier [124]. Filaggrin components, known for their anti-Staphylococcus aureus (*S. aureus*) properties, are often depleted in CTCL, making skin lesions vulnerable to colonization, particularly by *S. aureus*, the most common pathogen in CTCL-related skin infections [116,121,125,126]. Importantly, the risk of skin colonization by *S. aureus* increases with disease progression, and between one-third and two-thirds of patients with advanced disease harbor *S. aureus*, a large proportion of which produce toxins such as staphylococcal enterotoxins (SEs) [121,125]. This colonization not only poses a risk for invasive infections but also appears to exacerbate disease activity and potentially contributes to drug resistance in malignant T cells [120,127,128].

Antibiotic therapy often provides temporary relief by reducing skin symptoms and improving disease status; however, the recurrence of *S. aureus* colonization is common [129,130,131,132,133]. Aggressive antibiotic treatment targeting toxin-producing *S. aureus* strains has shown promising results, leading to reductions in skin symptoms, disease activity, and the number of malignant T cells in skin lesions [130]. This supports the notion that *S. aureus* colonization plays a pivotal role in fueling disease progression in CTCL [120]. Because many patients harbor methicillin-resistant *S. aureus* (MRSA) [132], and lesional skin becomes recolonized shortly after the antibiotic treatment is halted [131], new (antibiotic-free) treatments are warranted, such as engineered bacteriophage-derived endolysins, which selectively kills enterotoxin-producing *S. aureus* isolated from CTCL skin lesions [134].

In addition to a compromised skin barrier as a likely port of entrance for *S. aureus* and other bacteria implicated in CTCL [124,135], there is also evidence suggesting a potential deficiency in antimicrobial defense mechanisms such as an impaired expression of antimicrobial peptides including cathelicidin and other molecules involved in the antibacterial defense in the skin [135,136,137,138]. As mentioned above, cathelicidin is a potent antimicrobial peptide, which targets an array of bacteria including *S. aureus*. As cathelicidin expression is compromised in lesional skin in CTCL patients [138] and is induced by vitamin D in healthy individuals [27,139], it is possible that a deficient expression is due to a deficient vitamin D synthesis in affected skin or, alternatively, a deficient response to vitamin D in CTCL skin lesions.

## 5. Vitamin D in CTCL

For decades, vitamin D has been suspected to play a role in the pathogenesis of CTCL [94]. Concerning the vitamin D nomenclature, it is known that it is the active form of vitamin D, 1,25(OH)_2_D, that exerts the biological effects of vitamin D. However, in the literature, the term vitamin D is generally used without discrimination between the various forms of vitamin D. We will follow the same nomenclature for vitamin D in this review if not otherwise stated. The most common variant, MF, often presents in light-protected skin areas devoid of sun-induced vitamin D, which early on prompted the hypothesis that sun-induced vitamin D expression could play a role in the repression of the disease, and inversely, that low levels of vitamin D were permissive for the development of MF [28,140]. In support of this idea, it was reported that a history of greater sun exposure was associated with some degree of protection from contracting some subtypes of non-Hodgkin lymphoma, including CTCL [141,142], and recently, high sun exposure was associated with a lower risk of developing early-stage MF in Caucasians [106]. This notion also gained some indirect support from the observations that the disease incidence increased with distance from the equator for several mature cutaneous T-cell neoplasms, including MF/Sezary syndrome [29], and the observation that greater body surface area involvement was inversely correlated with vitamin D levels in MF patients [28]. Likewise, the beneficial response to phototherapy in CTCL patients has also been ascribed to vitamin D [85], although a multitude of vitamin D-independent mechanisms may also be involved in the clinical effects of phototherapy [143,144]. In addition, vitamin D and vitamin D analogs seem to potentiate the in vitro effect of chemotherapeutic agents such as doxorubicin, sometimes used in the treatment of advanced-stage CTCL patients [145].

Genetic factors involving the VDR have previously been suggested to play a role in CTCL pathogenesis [146,147,148]. One study obtained evidence that a single-nucleotide polymorphism in the VDR-Fokl gene was associated with CTCL [146], whereas other studies did not find this association [147,148]. On the contrary, Incel et al. [147] reported that a single-nucleotide polymorphism in VDR Taq1 was associated with decreased susceptibility to MF. Notably, a recent study of CTCL in a larger cohort of twins found that the disease was not inherited and that genetic predisposition did not play a major role in CTCL [123].

## 6. Vitamin D Serum Levels in CTCL

A few studies have examined vitamin D levels in CTCL patients compared to the background population, patients with other cancers, and patients with benign inflammatory diseases [28,146,148,149,150]. Some studies reported significant reductions in the serum levels of vitamin D in CTCL patients when compared to the general population and/or matched controls [28,141,149,150], whereas others did not [148]. Notably, the serum levels of vitamin D in CTCL patients were comparable to the serum levels observed in comparable groups of patients with benign dermatoses such as atopic dermatitis and psoriasis [149]. Thus, a compromised vitamin D expression seemed to be due to the inflammatory process rather than the cancer per se.

## 7. Vitamin D in the Pathogenesis of CTCL

**Figure 2:**
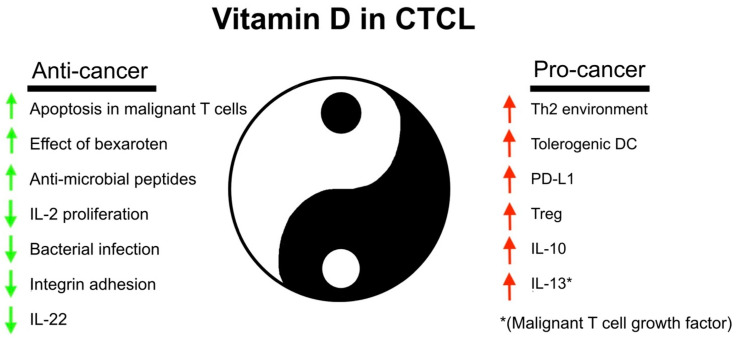
The many anti- and pro-cancer roles of vitamin D in CTCL. IL, interleukin; Th2, T helper 2 cell; DC, dendritic cell; PD-L1, programmed death ligand-1; Treg, regulatory T cell.

Some studies have addressed the expression of the VDR and the effect of vitamin D in malignant T cells, and they report that primary malignant T cells and T-cell lines express the VDR and respond to vitamin D in relevant doses [150,151]. Notably, vitamin D triggered apoptosis in both primary Sezary syndrome malignant T cells and an MF-derived, malignant T-cell line [150]. Moreover, vitamin D inhibited the spontaneous and induced expression of IL-22 mRNA and protein by malignant T cells [74]. The inhibition was mediated via a repressive vitamin D response element in the IL-22 promoter and independently of JAK/STAT signaling [74], which is constitutively active in malignant T-cell lines [152]. As IL-22 is a survival factor in malignant T cells and is believed to play a role in the pathogenesis through changes in the TME [153], these findings suggest a potential anticancer effect of vitamin D both directly through the inhibition of survival signals in malignant T cells and indirectly through the modulation of the TME (Figure 2, left side). Mrotzek et al. [150] examined the effect of vitamin D on the proliferation of an MF cell line treated with bexarotene routinely used in the treatment of CTCL patients [94] and found that vitamin D inhibited the proliferation of bexarotene-treated cells showing synergistic anticancer effects of vitamin D and bexarotene. In addition, vitamin D inhibits integrin-mediated adhesion by malignant T-cell lines and interactions with other cells in the TME [154], which are believed to promote the proliferation of malignant T cells [155]. Thus, vitamin D may inhibit disease activity via direct effects on the malignant T cell, an increase in sensitivity to treatment, and/or indirectly via the inhibition of interactions with the TME.

It is well established that CTCL patients have an increased susceptibility to bacterial skin infection, which is likely due to a compromised skin barrier and deregulated expression of antimicrobial peptides [124]. As mentioned above, vitamin D plays an important role in the expression of antimicrobial peptides as illustrated by an increased risk of bacterial infections in patients with severe vitamin D deficiency [156,157,158] and genetic conditions such as patients with loss-of-function mutations in the VDR [67]. Therefore, it is possible—but remains to be proven—that vitamin D deficiency could predispose to an increased risk of bacterial skin infections in CTCL patients, and inversely that vitamin D—alone or in combination with antibacterial proteins such as endolysins [134]—could have a preventive effect on bacterial infections in these patients. As bacteria such as enterotoxin-producing *S. aureus* seem to fuel disease activity and drug resistance in patients with advanced disease [128,130], vitamin D may inhibit disease activity via the induction of antimicrobial peptides (Figure 2, left side).

Most of the symptoms related to CTCL, such as itch, pain, and skin lesions, are the results of inflammatory processes [91,155]. As vitamin D has anti-inflammatory properties [72], it is possible that vitamin D could alleviate inflammation-associated symptoms. On the other hand, vitamin D also promotes a series of immunological changes, which could promote—rather than inhibit—symptoms and disease progression. First, vitamin D enhances Th2 immune responses [24,52,53,55]. As the Th2 cytokines IL-4 and IL-13 are growth factors for malignant T cells and induce the repression of filaggrin in the skin [124,159], vitamin D could indirectly enhance malignant proliferation, disease progression, and the deterioration of the skin barrier (Figure 2, right side). Furthermore, vitamin D inhibits Th1 responses and IL-2-driven proliferation while promoting Treg, tolerogenic macrophages, and dendritic cells, as well as the expression of immune checkpoint molecules such as PD-L1 and PD-L2 [16,21,160,161,162,163,164,165,166], suggesting that vitamin D may also inhibit anticancer immunity (Figure 2, right side). Taken together, these seemingly opposing effects described above indicate that vitamin D may have both beneficial and harmful effects depending on the context, suggesting a complex role of vitamin D in CTCL biology (Figure 2).

## 8. Effects of Vitamin D Treatment in CTCL

In an early case study, Scott-Mackie et al. [167] were the first to report on the beneficial effect of treatment with topical calcipotriol in a patient with low-grade angioimmunoblastic T-cell lymphoma. Shortly after, French et al. [168] reported that systemic therapy with calcipotriol in combination with acitretin was associated with a clearing of the skin lesions in a patient with CTCL, whereas topical treatment with calcipotriol had no effect. These studies prompted more investigations, but other researchers did not replicate these initial, positive findings. On the contrary, later case studies reported on disease aggravation and progression following treatment with vitamin D in combination with the retinoids acitretin [169] and isotretinoin [170]. Thus, in one patient, new cutaneous lesions and an extra-cutaneous lesion appeared and rapidly increased during treatment with acitretin and calcitriol [169], whereas three patients experienced rapidly increasing skin lesions and tumors following the initiation of treatment with calcitriol and isotretinoin [170], prompting warnings and caution in relation to the use of calcitriol in CTCL. Twenty years later, the first systematic prospective study addressing vitamin D status and the effect of vitamin D supplementation in a larger cohort of 311 CTCL patients was performed [28]. As mentioned above, most patients displayed vitamin D deficiency as judged by the serum levels of vitamin 25(OH)D, which was, however, not significantly different from that observed in comparable patients with other types of cancer. Importantly, the correction of vitamin D deficiency was successful in a large fraction of patients (27–50% depending on the vitamin D treatment regimen). Because the disease course and responses to standard CTCL therapy were similar among patients with corrected and persistently low levels of vitamin D, it was concluded that the correction of vitamin D deficiency did not affect clinical disease activity, response to treatment, or disease course in CTCL patients [28].

## 9. Phototherapy and Sunlight Modulate Skin Inflammation in Vitamin D Independent Ways

It was the common observation of a “bathing suit” distribution (i.e., skin areas usually protected from sunlight) of skin lesions in MF patients that led to the assumption that sunlight and phototherapy induced its effects through vitamin D production in the skin. However, sunlight and phototherapy affect the skin in multiple ways, several of which are independent of vitamin D. For instance, UV light stimulates DNA damage, platelet-activating factor, the activation of the aryl hydrocarbon receptor, and oxidative stress-related enzymes, all of which, like vitamin D, subsequently stimulate regulatory mechanisms via Treg and Langerhans cells and factors like IL-10, TGFβ, and IDO-1 [84,143,144]. Notably, phototherapy was recently shown to restore a deficient type I IFN production and enhance antitumor responses in MF, providing an alternative explanation for the effects of phototherapy in CTCL [171].

## 10. Concluding Remarks

The longstanding question concerning the role of vitamin D in the pathogenesis, treatment, and prevention of CTCL remains largely unresolved. Although some epidemiological data suggest a protective role of sunlight, and experimental studies indicate anticancer effects, other data suggest that vitamin D may also have tumor-promoting effects. Likewise, clinical studies provide opposing results or no effect at all. Taken together, these data indicate a complex and incompletely understood role of vitamin D in CTCL that warrants further investigation. Importantly, they do not provide a rationale for vitamin D therapy beyond the personalized treatment of vitamin D deficiency in individual patients.
